# Sedation-Assisted, Non-sedated, and Escalated Removal of Pediatric ENT Foreign Bodies in Emergency Settings: A Systematic Review

**DOI:** 10.7759/cureus.114951

**Published:** 2026-08-21

**Authors:** Hassan H Al Bader, Hassan A Alalawi, Rahaf K Alhajji, Taif K H‎aka‎mi, Ahmed F Alluwaim, Muath H Alqesair, Liyan A Alwadai, Hussain M Reda, Sulaiman A Darbashi, Abdullah F Aljoudi, Yousef M Bajhzer

**Affiliations:** 1 Otolaryngology, King Faisal Hospital, Al-Ahsa, SAU; 2 General Practice, Prince Saudi Bin Jalawy Hospital, Al-Ahsa, SAU; 3 College of Medicine, University of Szeged Albert Szent-Györgyi Medical School, Szeged, HUN; 4 College of Medicine, King Khalid University, Abha, SAU; 5 Faculty of Medicine, Dar Al Uloom University, Riyadh, SAU; 6 Otolaryngology, Jazan General Hospital, Jazan Health Cluster, Jazan, SAU; 7 Faculty of Medicine, Umm Al-Qura University, Makkah, SAU

**Keywords:** ear foreign body, emergency department, foreign body removal, nasal foreign body, non-sedated removal, pediatric emergency medicine, procedural sedation, sedation-assisted removal, throat foreign body

## Abstract

Pediatric ear, nose, and throat (ENT) foreign bodies are among the most common procedural challenges in emergency medicine. Substantial uncertainty exists regarding the optimal approach to removal, including sedation-assisted versus non-sedated techniques, and the appropriate thresholds for escalation to otolaryngology or the operating room.

A systematic literature search was conducted in PubMed, the Cochrane Library, Web of Science, and Scopus (search date: June 2026). Studies were included if they reported outcomes of ENT foreign body removal in pediatric or predominantly pediatric emergency and acute care settings. Risk of bias was assessed using domain-specific methodological appraisal. Meta-analysis was not performed because of substantial clinical and methodological heterogeneity.

Eight studies published between 2004 and 2022 were included from the United States, Singapore, Australia, and Israel, representing 2,332 patients and 2,336 removal attempts or study-level encounters. The difference reflects one study that reported 254 removal attempts involving 250 children. Removal success varied according to clinical setting and case complexity. In one pediatric ED cohort, successful removal was achieved in 204/254 (80.3%) ear foreign body attempts, while another study reported first-attempt success of 82.4% for pediatric emergency physicians and 96.1% for ENT physicians. Sedation was used in 1/254 (0.4%) to 71/312 (22.8%) of cases and was generally reserved for difficult or uncooperative patients or after failed non-sedated attempts, precluding causal comparison between strategies. Reported complication rates varied substantially because of differences in definitions and patient selection; one study reported complications in 30/254 (11.8%) cases, whereas another reported complications in 40/380 (10.5%), including tympanic membrane perforation in 1/380 (0.3%). Referral and escalation also varied according to clinical setting and case complexity. In one study, 38/275 (13.8%) patients were referred to ENT after previous removal attempts, while in another study, 44.8% were referred to an ENT clinic for further assessment. In one study, operative intervention was required in 2/44 (4.6%) patients initially managed by ENT and 13/38 (34.2%) patients referred to ENT after previous unsuccessful attempts; whereas in another study, 51/380 (13.4%) underwent removal in the operating theatre under general anaesthesia. ED procedural sedation demonstrated a safety profile comparable to operating-room management while substantially reducing costs. Repeated removal attempts and difficult foreign body characteristics were consistently associated with failure, complications, or escalation of care.

Overall, emergency physicians can successfully remove most pediatric ear and nasal foreign bodies, although success varies substantially according to clinical setting and case complexity. Sedation appears to be a useful escalation strategy for uncooperative children or after failed initial attempts, although current evidence does not support a causal comparison with non-sedated approaches. Early otolaryngology referral is appropriate after failed attempts, for difficult foreign bodies, suspected tympanic membrane involvement, or button battery exposure. High-quality prospective comparative studies using standardized outcome definitions are needed to strengthen the evidence base.

## Introduction and background

Foreign bodies (FBs) in the ear, nose, and throat (ENT) are common causes of emergency and acute-care presentations among children. Children aged two to eight years frequently insert objects such as beads, toy components, food materials, paper, cotton, insects, and household items into the ear canal, nasal cavity, or oropharynx [1-3]. Although most cases are not immediately life-threatening, successful management depends on adequate visualization, appropriate equipment, provider expertise, and patient cooperation. Delayed recognition or unsuccessful removal may result in infection, mucosal or canal trauma, airway compromise, or chemical injury, particularly in cases involving button battery ingestion or insertion [3-7].

The difficulty of foreign body removal varies according to anatomical location, object characteristics, depth, accessibility, and the child’s ability to cooperate. Foreign bodies in the external auditory canal (EAC) can be particularly challenging because the medial bony canal is narrow and sensitive, making instrumentation more likely to cause pain, bleeding, canal injury, and further loss of cooperation [1-4]. Smooth or firmly lodged objects, such as beads and stones, are associated with increased removal difficulty. Repeated unsuccessful attempts may worsen swelling, reduce visibility, and increase the likelihood of complications or the need for specialist intervention [2,5].

A key management decision in emergency practice is whether to attempt removal in the emergency department (ED) or refer directly to an otolaryngologist. Many pediatric ED providers can successfully remove uncomplicated foreign bodies; however, early specialist involvement is often appropriate for cases involving failed previous attempts, difficult-to-grasp objects, medial canal location, suspected tympanic membrane injury, button batteries, or severe patient distress [1-6]. Although specialist-managed cases often demonstrate higher success rates, these comparisons are influenced by referral patterns because more complex cases are preferentially managed by otolaryngologists [1,2].

Procedural sedation is an important escalation option for children in whom non-sedated removal is unsuccessful, anticipated to fail, or considered unsafe because of inadequate cooperation. Sedation may improve procedural conditions by reducing pain, anxiety, and movement, thereby facilitating safer removal [3-7]. However, pediatric procedural sedation requires trained personnel, appropriate monitoring, airway preparedness, and recovery resources. Importantly, sedation is generally used selectively for more challenging cases rather than as a standardized first-line approach, creating difficulties when comparing outcomes between sedated and non-sedated removal strategies [7-14].

Despite the frequency of pediatric ENT foreign body presentations, evidence guiding management remains heterogeneous. Existing studies differ in anatomical focus, patient populations, clinical settings, provider experience, sedation indications, and outcome definitions [3-7]. Furthermore, because sedation is typically reserved for complex or failed cases, direct comparisons between sedation-assisted and non-sedated removal may be affected by selection bias. These variations limit the ability to combine results quantitatively and highlight the need for a structured synthesis of available evidence.

This systematic review aims to evaluate management strategies for pediatric ENT foreign bodies in emergency and acute-care settings, including non-sedated removal success, sedation-assisted outcomes, specialist referral, operative management or general anaesthesia, complications, predictors of failure or escalation, and resource utilization.

## Review

Methods

Design and Reporting Framework

This systematic review was conducted and reported according to the Preferred Reporting Items for Systematic Reviews and Meta-Analyses (PRISMA 2020) statement [15]. Because substantial clinical and methodological heterogeneity prevented appropriate meta-analysis, findings were presented using structured tables, study-level event counts, proportions, and narrative synthesis.

Search Strategy

A systematic search was conducted in PubMed, the Cochrane Library, Web of Science, and Scopus in June 2026. Search terms combined concepts related to pediatric ENT foreign bodies, emergency and acute-care settings, removal procedures, and sedation or anesthesia. The search included both controlled vocabulary and free-text terms covering foreign bodies, pediatric populations, anatomical sites, emergency care, procedural sedation, sedative agents, and anesthesia. No restrictions were applied regarding language or publication date. However, study designs were limited according to the predefined eligibility criteria, and only studies reporting extractable outcome data relevant to pediatric ENT foreign body removal were considered eligible.

Eligibility Criteria

Studies were eligible if they included pediatric patients (aged <18 years) with foreign bodies involving the external auditory canal, nasal cavity, or pharynx/oropharynx. Eligible settings included EDs, pediatric emergency departments, urgent care, or other acute-care settings. Studies were included if they reported at least one relevant outcome: removal success, sedation use, complications, ENT referral, escalation to the operating room or general anesthesia, predictors of failure or escalation, or cost and resource outcomes.

Eligible study designs included observational cohorts, comparative studies, and case series with extractable outcome data. Case reports with fewer than five patients, reviews without primary data, editorials, commentaries, and studies involving only adults without separable pediatric data were excluded.

Study Selection

Search results were imported into a reference management system, and duplicates were removed. Titles and abstracts were screened, followed by full-text assessment of potentially eligible studies. Reasons for full-text exclusion were recorded, and the selection process was summarized using a PRISMA 2020 flow diagram.

Data Extraction

Data were extracted using a standardized form. Extracted variables included study characteristics, country, design, clinical setting, sample size, patient age, anatomical site, provider type, sedation approach, removal success rates, first-attempt success, complications, ENT referral, escalation to operative management or general anesthesia, predictors of failure or adverse outcomes, and cost or resource-use data.

When reported percentages differed from available numerator and denominator values, both were recorded, and discrepancies were noted. Studies with important sampling or reporting limitations were flagged during synthesis.

Risk of Bias Assessment

Risk of bias was assessed using the Risk Of Bias In Non-randomized Studies of Interventions (ROBINS-I) tool [16]. ROBINS-I was selected because all included studies were observational and evaluated non-randomized clinical management strategies, including sedation-assisted removal, non-sedated removal, and escalation to specialist or operative management.

The ROBINS-I framework assesses bias across seven domains. Each domain was judged as Low risk, Moderate risk, Serious risk, Critical risk, or No information, according to ROBINS-I guidance. Particular consideration was given to confounding by indication because sedation was generally reserved for younger, less cooperative children or those with failed previous removal attempts. Referral-based cohorts were also considered at increased risk of selection bias because more complex cases were preferentially managed by otolaryngology services. No study was excluded solely because of risk of bias. The results of the appraisal were used to inform interpretation of the narrative synthesis.

Synthesis Without Meta-Analysis

Meta-analysis was not performed because of substantial heterogeneity in study populations, anatomical sites, clinical settings, provider types, sedation indications, outcome definitions, and reporting methods. Sedation was frequently used selectively for complex cases or after failed attempts, limiting direct comparisons with non-sedated removal groups. Findings were summarized using study-level counts, proportions, ranges, and narrative comparisons. Quantitative pooling and heterogeneity statistics were not calculated.

Results

Study Selection

**Figure 1:**
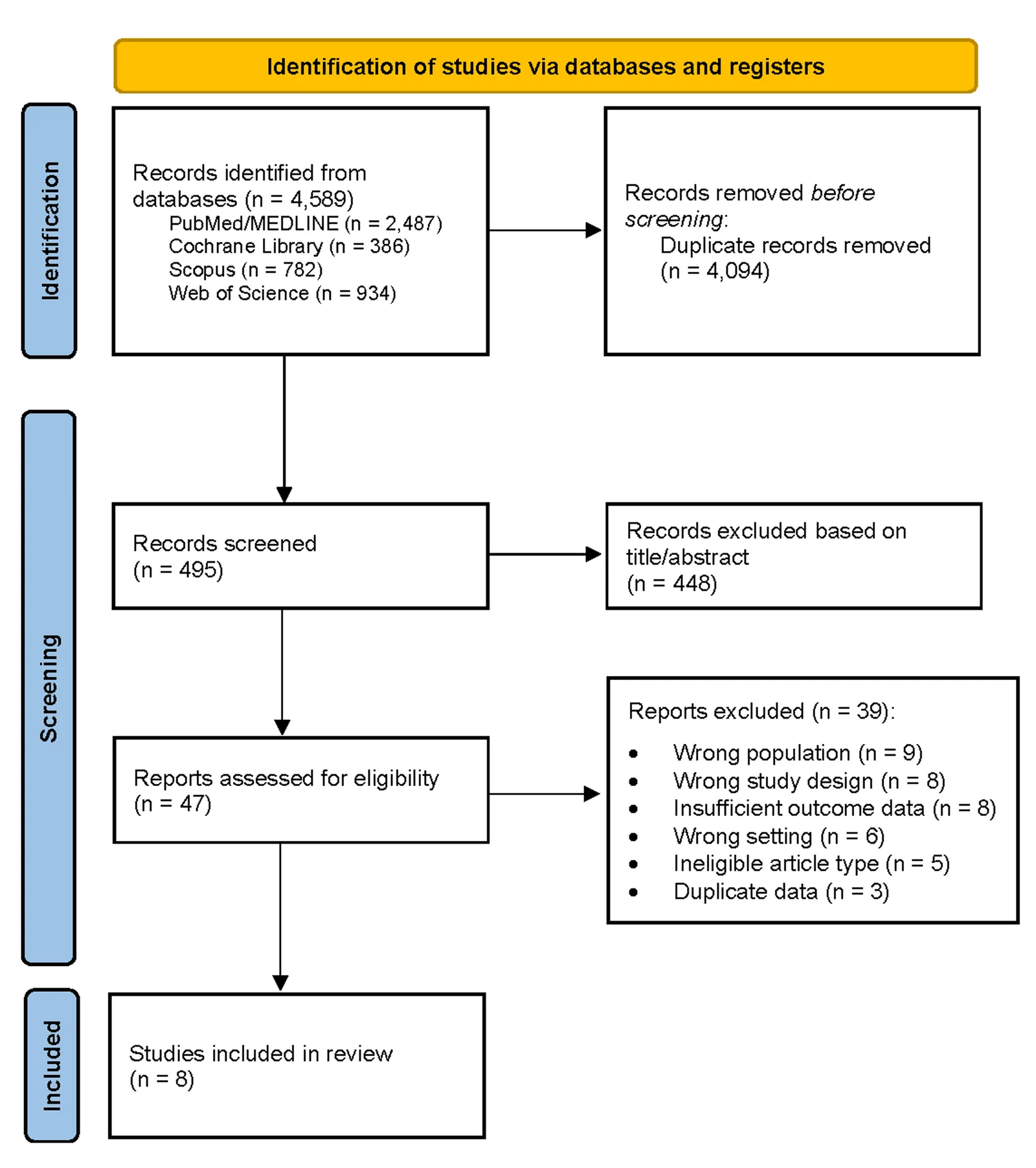
PRISMA flow diagram depicting the study selection process for the systematic review PRISMA (Preferred Reporting Items for Systematic Reviews and Meta-Analyses) flow diagram detailing the study selection process for this systematic review. Following the identification of records through database searching and other sources, duplicates were removed, and the remaining records were screened. Full-text articles were assessed for eligibility, with exclusions documented along with reasons. Studies meeting the inclusion criteria were included in the final synthesis.

The database search identified 4,589 records, of which 4,094 duplicates were removed. After title and abstract screening of 495 records, 47 full-text articles were assessed for eligibility. Thirty-nine studies were excluded following full-text review, leaving eight studies for inclusion in the systematic review. All studies contributed to the narrative synthesis, although eligibility for individual outcome analyses varied according to study population, anatomical site, clinical setting, and reported outcomes (Figure 1).

Study Characteristics

Eight retrospective observational studies, published between 2004 and 2022, were included, representing approximately 2,332 patient encounters from the United States, Singapore, Australia, and Israel [1-8]. Six studies evaluated ear foreign bodies exclusively [2,3,4,6-8], one included both ear and nasal foreign bodies [1], and one examined ear, nasal, and throat foreign bodies [5]. Five studies were restricted to pediatric patients [1,3,5,7,8], whereas three included mixed-age populations [2,4,6]. Most studies were conducted in tertiary EDs, although some also included primary care physicians or ENT services. Sample sizes ranged from 63 to 495 patients (Table 1).

**Table 1:** Characteristics of included studies This table summarizes the demographic, methodological, and clinical characteristics of the included studies, including study setting, sample size, patient age, anatomical sites of foreign bodies, and key study-specific considerations relevant to interpretation. Values are presented as reported in the original studies. Some studies included mixed-age populations, mixed clinical settings, or referral-based cohorts; therefore, reported sample sizes and characteristics should be interpreted in the context of each study design. The Olson study represents a pre-selected sedation cohort and is not representative of the overall emergency department population. ED = emergency department; ENT = ear, nose, and throat; PCP = primary care physician; PED = pediatric emergency department; UC = urgent care.

Study ID	Country	Design	Setting	N	Age	Site(s)	Key flags	Main conclusions
Brown et al. 2004 [1]	USA	Retrospective chart review	Tertiary university ED	312 (174 ear, 138 nose)	<18 years; ear median 6.1 years	Ear + nose	Sedation after failed attempts; 23% sedation rate; ketamine 92%	Procedural sedation was used selectively after unsuccessful or difficult removal attempts and facilitated successful removal of pediatric ENT foreign bodies in the ED.
Marin et al. 2006 [2]	USA	Retrospective case series	Tertiary pediatric ED	250 children; 254 removal attempts	2 months–20 years; mean 6 years 7 months	Ear only	Teaching hospital; 0.4% sedated; 19% referred from outside	Most pediatric ear foreign bodies were successfully removed in the ED, with sedation rarely required. Multiple attempts and difficult objects increased the risk of complications or failure.
Mingo et al. 2020 [3]	USA	Retrospective chart review	Tertiary hospital (multi-setting)	275	1–18 years	Ear only	ED 133, PCP 60, ENT 44, secondary ENT 38; may include transfers	Removal success differed between specialties, with higher success in ENT-managed cases; however, referral patterns and case complexity influenced comparisons.
Ng et al. 2018 [4]	Australia	Retrospective review	Major hospital ED	380	1–85 years; median 21 years	Ear only	Non-consecutive; 49% ≤20 years; no separate pediatric tables	Repeated attempts and challenging foreign body characteristics were associated with increased failure and complications during aural foreign body removal.
Ngo et al. 2005 [5]	Singapore	Retrospective	Tertiary pediatric ED	353 (117 ear, 125 nose, 111 throat)	<16 years; mean 4.73 years	Ear + nose + throat	Mixed ENT sites; mixed ED and ENT providers; ambiguous denominators	Pediatric ENT foreign bodies were commonly managed successfully, although outcomes varied by anatomical site and provider involvement.
Olson et al. 2018 [6]	USA	Retrospective chart review	Tertiary ED (Mayo Clinic)	63 (sedation subset of 495)	2.2–46.8 years; median 5.3 years	Ear only	Pre-selected sedation cohort; not a general ED cohort	ED procedural sedation for ear foreign body removal was safe and less costly than operating room management, supporting sedation as an alternative for selected difficult cases.
Shih et al. 2021 [7]	USA	Retrospective chart review	Tertiary children’s hospital + satellites	366	1–16 years; mean 6.49 years	Ear only	Multi-setting (ED/UC 63, PCP 230, ENT 183); high referral bias in ED/UC	Success rates varied by clinical setting, with higher success in specialist-managed cases; differences were influenced by referral patterns and case selection.
Weksler et al. 2022 [8]	Israel	Retrospective analytical comparison	Tertiary pediatric ED	333	Mean 6.53 years; median 5 years	Ear only	77.8% managed by ENT; PED first attempt 22.2%; all sedation by ENT	ENT management achieved high removal success; emergency physician first-attempt success was lower but improved when patients with previous failed attempts were excluded.

Risk of Bias

Risk of bias was assessed using the ROBINS-I tool [16]. All included studies were retrospective observational studies and were therefore considered susceptible to bias related to non-randomized treatment allocation, confounding, and selection mechanisms. Common methodological limitations included single-centre designs, referral-based sampling, heterogeneous patient populations, inconsistent outcome definitions, and incomplete reporting of provider experience and procedural details [1-8]. Sedation was generally reserved for children with difficult foreign bodies, poor cooperation, or failed initial removal attempts, resulting in substantial confounding by indication and limiting direct comparisons between sedation-assisted and non-sedated approaches. Studies involving otolaryngology referral populations were also considered at increased risk of selection bias because more complex cases were preferentially managed by specialist services, reducing comparability with general emergency department cohorts (Table 2).

**Table 2:** Risk of bias assessment of included studies using the ROBINS-I tool ROBINS-I = Risk Of Bias In Non-randomized Studies of Interventions. Domains were assessed according to ROBINS-I guidance and categorized as Low risk, Moderate risk, Serious risk, Critical risk, or No information [16]. Overall risk of bias was determined according to the highest level of concern identified across domains, with particular consideration given to confounding and selection bias because sedation and specialist referral were preferentially used for more complex cases.

Study ID	Bias due to confounding	Bias in selection of participants into the study	Bias in classification of interventions	Bias due to deviations from intended interventions	Bias due to missing data	Bias in measurement of outcomes	Bias in selection of the reported result	Overall risk of bias
Brown et al. 2004 [1]	Serious	Moderate	Low	Moderate	Moderate	Moderate	Moderate	Serious
Marin et al. 2006 [2]	Serious	Moderate	Low	Moderate	Moderate	Moderate	Moderate	Serious
Mingo et al. 2020 [3]	Serious	Serious	Moderate	Moderate	Low	Moderate	Moderate	Serious
Ng et al. 2018 [4]	Serious	Serious	Moderate	Moderate	Moderate	Moderate	Moderate	Serious
Ngo et al. 2005 [5]	Serious	Serious	Moderate	Moderate	Serious	Moderate	Moderate	Serious
Olson et al. 2018 [6]	Critical	Serious	Low	Moderate	Low	Moderate	Moderate	Critical
Shih et al. 2021 [7]	Serious	Serious	Moderate	Moderate	Moderate	Moderate	Moderate	Serious
Weksler et al. 2022 [8]	Serious	Serious	Low	Moderate	Moderate	Moderate	Moderate	Serious

Removal Success

Six studies reported removal success for ear foreign bodies [1-4,7,8]. In the Brown et al. study, emergency physicians successfully removed 84% of ear foreign bodies and 95% of nasal foreign bodies; the study included 174 ear and 138 nasal foreign bodies, respectively [1]. In the Marin et al. study, emergency physicians successfully removed 204/254 (80.3%) ear foreign bodies, with failure or referral required for the remaining cases [2]. In the Mingo et al. study, successful retrieval was achieved in 95.4% of patients initially managed by ENT, 85.7% of those initially managed in the ED, 75.0% of those managed by primary care physicians, and 65.8% of those referred to ENT after previous unsuccessful attempts [3]. These groups comprised 44, 133, 60, and 38 patients, respectively [3]. In the Weksler et al. study, first-attempt removal was successful in 82.4% of cases managed by pediatric emergency physicians and 96.1% of cases managed by ENT physicians; after excluding patients with previous removal attempts, success rates were 93.9% and 96.8%, respectively [8].

Ng et al. reported successful first-attempt removal in 278/380 (73.2%) patients with aural foreign bodies [4]. Because this study included patients aged 1-85 years and did not provide separate pediatric outcome tables, its findings were not used to estimate pediatric-specific success rates [4].

Three studies evaluated first-attempt success [3,4,8]. ENT physicians generally achieved higher crude first-attempt success than emergency or primary-care physicians; however, the difference was reduced after excluding patients with previous unsuccessful removal attempts [3,8]. Because the studies differed in patient selection, clinical setting, and outcome definitions, quantitative pooling was not performed.

Sedation-Assisted and Non-sedated Removal

Seven studies reported sedation use [1-6,8]. In Brown et al., procedural sedation was used in 71/312 (22.8%) children with single-orifice foreign bodies, including 43/174 (24.7%) with ear foreign bodies and 28/138 (20.3%) with nasal foreign bodies; ketamine accounted for 92% of sedations [1].

In Marin et al., sedation was required in 1/254 (0.4%) ear foreign body removal attempts [2]. In Ng et al., 20/380 (5.3%) patients underwent removal in the ED with sedation, whereas 309/380 (81.3%) underwent removal in the ED without sedation and 51/380 (13.4%) underwent removal in the operating theatre under general anesthesia [4].

Sedation was generally used as an escalation strategy for difficult or uncooperative patients or after failed non-sedated attempts rather than as first-line management [1,3,4,8]. In Brown et al., more than half of patients receiving sedation had previously undergone unsuccessful attempts without sedation by the same physician [1]. In Ng et al., patients undergoing sedated ED removal had a median age of 4 years compared with 30 years among patients undergoing non-sedated ED removal, consistent with selective use of sedation in younger or more difficult cases [4].

Complications and Safety

Reported complication rates varied substantially between studies because of differences in definitions and patient selection. Marin et al. reported complications in 30/254 (11.8%) cases, with multiple removal attempts significantly associated with both failure and complications [2]. Ng et al. reported complications in 40/380 (10.5%) patients; these included canal abrasion in 33/380 (8.7%), bleeding in 6/380 (1.6%), an inflamed tympanic membrane in 1/380 (0.3%), and tympanic membrane perforation in 1/380 (0.3%) [4].

In the Mingo et al. study, minor complications occurred in 2.3%-6.0% of patients managed initially by ED or primary care providers, compared with 26.3% among patients referred to ENT after previous unsuccessful attempts; two tympanic membrane perforations occurred in the latter group [3]. The study also found that operative intervention was required in 4.6% of patients initially managed by ENT compared with 34.2% of those referred to ENT after previous unsuccessful attempts [3].

Shih et al. reported complications in 22.22% of ED/urgent-care attempts, compared with 2.61% of primary-care attempts; the authors emphasized that referral and case-selection patterns substantially influenced these comparisons [7].

Overall, the available evidence indicates that most reported complications were minor, whereas serious complications such as tympanic membrane perforation were uncommon but occurred more frequently after repeated or unsuccessful prior attempts [2-4,7,8].

Referral and Escalation of Care

Referral and escalation rates varied substantially according to study setting and case selection. In Mingo et al., 44/275 (16.0%) patients initially presented to ENT, 133/275 (48.4%) to the ED, 60/275 (21.8%) to a pediatrician, and 38/275 (13.8%) were referred to ENT after previous removal attempts [3]. Operative intervention was required in 2/44 (4.6%) patients initially managed by ENT compared with 13/38 (34.2%) patients referred to ENT after previous attempts [3].

In Ngo et al., an emergency physician attempted removal in 76.8% of 353 patients, corresponding to approximately 271/353 patients; ENT specialist referral directly from the ED occurred in 1.7% (approximately 6/353), 50.1% were discharged after successful ED removal, 4.2% were admitted for removal, and 44.8% were referred to the ENT clinic for further assessment [5]. Because the published percentages were reported without exact numerators for these outcomes, the percentages are retained as reported rather than converting them to potentially imprecise whole-number counts.

In Ng et al., 51/380 (13.4%) patients ultimately underwent removal in the operating theatre under general anesthesia, while 20/380 (5.3%) underwent ED removal with sedation and 309/380 (81.3%) underwent ED removal without sedation [4].

Predictors of Failure and Complications

Repeated removal attempts were consistently associated with failure and complications. Marin et al. found that multiple attempts were associated with failed removal (RR, 6.0; 95% CI, 3.0-12.0) and complications (RR, 3.1; 95% CI, 1.5-6.3) [2]. Ng et al. similarly found that increasing numbers of removal attempts predicted complications, with three attempts associated with an odds ratio of 3.4 (95% CI, 1.03-11.16), four attempts with an odds ratio of 4.8 (95% CI, 1.65-20.25), and five attempts with an odds ratio of 11.6 (95% CI, 2.85-46.8) [4].

Mingo et al. found that patients referred to ENT after previous unsuccessful attempts had both lower retrieval success and higher rates of minor complications and operative intervention than patients initially managed by ENT [3]. Difficult-to-grasp foreign bodies and younger age were also associated with poorer outcomes, particularly among patients requiring referral after failed attempts [3].

Cost and Resource Use

Only Olson et al. specifically evaluated cost and safety of procedural sedation [6]. The study identified 63 patients requiring sedation for ear foreign body removal and found no appreciable difference in safety or otologic outcomes between emergency department conscious sedation and operating-room anesthesia. Operating-room management was associated with substantially greater costs, supporting ED conscious sedation as a potentially lower-cost alternative for appropriately selected patients [6].

Overall Synthesis

Across the included studies, removal success varied according to clinical setting and case complexity. Brown et al. reported successful removal of 84% of ear foreign bodies and 95% of nasal foreign bodies in the ED, while Marin et al. reported successful ED removal in 204/254 (80.3%) ear foreign body attempts [1,2]. Mingo et al. reported successful retrieval in 114/133 (85.7%) patients initially managed in the ED, 57/60 (95.0%) managed by primary care physicians, 42/44 (95.5%) initially managed by ENT, and 25/38 (65.8%) referred to ENT after previous unsuccessful attempts [3]. Weksler et al. reported first-attempt success rates of 82.4% for pediatric emergency physicians and 96.1% for ENT physicians, increasing to 93.9% and 96.8%, respectively, after exclusion of patients with previous removal attempts [8].

Sedation was generally reserved for selected difficult cases, with reported use ranging from 1/254 (0.4%) in Marin et al. to 71/312 (22.8%) in Brown et al. [1,2]. Serious complications were uncommon, although complication rates varied considerably across studies because of differences in definitions, referral patterns, and case complexity. Repeated or unsuccessful prior removal attempts were consistently associated with lower success and higher complication rates.

Limitations

This review has several limitations. All included studies were retrospective observational studies, mostly conducted at single tertiary or quaternary centers, limiting generalizability to other clinical settings. Considerable heterogeneity existed in patient populations, anatomical sites, outcome definitions, and reporting methods, particularly for complications and success rates, limiting direct comparisons between studies. Sedation was primarily used in more complex or previously failed cases, resulting in confounding by indication and preventing conclusions regarding the comparative effectiveness of sedation-assisted versus non-sedated removal. Some studies included mixed-age populations or combined different foreign body locations, requiring cautious interpretation of pediatric-specific findings. Additionally, the review was not prospectively registered, and grey literature and non-English studies were not systematically searched, which may have introduced publication and selection bias.

## Conclusions

Pediatric emergency clinicians can manage many ENT foreign bodies successfully when cases are appropriately selected, and removal is performed with careful technique and appropriate preparation. Procedural sedation remains an important escalation option for selected children who cannot tolerate non-sedated removal or when safe visualization and instrumentation cannot otherwise be achieved. However, the available evidence does not allow definitive conclusions regarding the comparative effectiveness of sedation-assisted versus non-sedated approaches because of important methodological limitations. Clinical factors such as unsuccessful prior attempts, challenging foreign body characteristics, and limited accessibility should guide early consideration of specialist involvement. Future prospective multicentre studies using standardized definitions and consistent outcome measures are needed to strengthen the evidence base and support the development of evidence-based management pathways.

## References

[1] Brown L, Denmark TK, Wittlake WA, Vargas EJ, Watson T, Crabb JW (2004). Procedural sedation use in the ED: management of pediatric ear and nose foreign bodies. Am J Emerg Med.

[2] Marin JR, Trainor JL (2006). Foreign body removal from the external auditory canal in a pediatric emergency department. Pediatr Emerg Care.

[3] Mingo K, Eleff D, Anne S, Osborne K (2020). Pediatric ear foreign body retrieval: A comparison across specialties. Am J Otolaryngol.

[4] Ng TT, Lim JW (2018). A 5-year review of aural foreign body removal in a major Victorian hospital. Aust J Otolaryngol.

[5] Ngo A, Ng KC, Sim TP (2005). Otorhinolaryngeal foreign bodies in children presenting to the emergency department. Singapore Med J.

[6] Olson MD, Saw J, Visscher SL, Balakrishnan K (2018). Cost comparison and safety of emergency department conscious sedation for the removal of ear foreign bodies. Int J Pediatr Otorhinolaryngol.

[7] Shih M, Brock L, Liu YC (2021). Pediatric aural foreign body extraction: comparison of efficacies among clinical settings and retrieval methods. Otolaryngol Head Neck Surg.

[8] Weksler CW, Heiman E, Weiser G (2022). Removal of external auditory canal foreign bodies in the pediatric emergency department - A retrospective comparison study. Int J Pediatr Otorhinolaryngol.

[9] Campbell M, McKenzie JE, Sowden A (2020). Synthesis without meta-analysis (SWiM) in systematic reviews: reporting guideline. BMJ.

[10] Schulze SL, Kerschner J, Beste D (2002). Pediatric external auditory canal foreign bodies: a review of 698 cases. Otolaryngol Head Neck Surg.

[11] Coté CJ, Wilson S (2019). Guidelines for monitoring and management of pediatric patients before, during, and after sedation for diagnostic and therapeutic procedures. Pediatrics.

[12] Davies PH, Benger JR (2000). Foreign bodies in the nose and ear: a review of techniques for removal in the emergency department. J Accid Emerg Med.

[13] White AC, Shih MC, Nguyen SA, Carol Liu YC (2023). Comparison of care settings for pediatric external auditory canal foreign bodies: a meta-analysis. Ann Otol Rhinol Laryngol.

[14] Olajide TG, Ologe FE, Arigbede OO (2020). Management of foreign bodies in the ear: a retrospective review of 123 cases in Nigeria [IN PRINT]. Ear Nose Throat J.

[15] Page MJ, McKenzie JE, Bossuyt PM (2021). The PRISMA 2020 statement: an updated guideline for reporting systematic reviews. Rev Esp Cardiol (Engl Ed).

[16] Sterne JA, Hernán MA, Reeves BC (2016). ROBINS-I: a tool for assessing risk of bias in non-randomised studies of interventions. BMJ.

